# Course of bipolar disorder in patients with Childhood maltreatment

**DOI:** 10.1192/j.eurpsy.2023.1450

**Published:** 2023-07-19

**Authors:** A. Mellouli, R. Masmoudi, F. Guermazi, F. Cherif, I. Feki, R. Sellami, J. Masmoudi

**Affiliations:** Psychiatry A Department, Hedi Chaker University Hospital, Sfax, Tunisia

## Abstract

**Introduction:**

Patients with mood disorders have the greater frequency of childhood trauma compared with the general population, and adverse childhood experiences can exert a negative impact on their clinical course. Therefore, many studies confirmed the relationship between childhood traumas and the disadvantageous features of the illness course.

**Objectives:**

The aim of this study was to determine the impact of negative childhood experiences on the clinical course of bipolar disorder.

**Methods:**

It was a cross-sectional descriptive and analytical study involving patients diagnosed with bipolar disorder and followed in the psychiatric department at the University Hospital of Sfax (Tunisia).Personal information form and Childhood trauma questionnaire (CTQ) were used for data acquisition. Euthymia was defined as a score on the Montgomery-Åsberg Depression Rating Scale (MADRS) not higher than 14 and by a score on the Young Mania Rating Scale (YMRS) not higher than seven.

**Results:**

We included 35 patients. Their mean age was 46.69 ± 12.01 years with a sex ratio (M/F)=0.45.

The average onset of bipolar disorder was 28.37±10.26 years and the average disease duration was 18.26 ± 11.55 years.

Almost the third of our population had a suicidal attempt (31.42%) and a violence history (28.57%). A family history of bipolar disorder was found in 57.14% of the patients.

The patients have been hospitalized at least once in 42.85% of cases.

Our patients have presented psychotic symptoms in 51.42% of cases and mixed characteristics in 57.14% of cases.

Emotional, physical and sexual abuse were reported by 42.85%, 37.14% , 31,42% of patients, respectively, while 74,28% and 42.85% of patients reported physical neglect and emotional neglect.

Early age at illness onset was significantly associated with total CTQ score (p=0.014) and the subtype sexual abuse (p=0.009). The presence of psychotic symptomswas significantly associated with total CTQ score (p=0.003) and emotional neglect (p=0.025). Physical neglect was associated with mixed characteristics (p=0.015). Emotional abuse was associated with a greater number of hospitalisations (p=0.023).

**Conclusions:**

Our results suggest that childhood trauma is associated with a more severe course of bipolar illness. Clinical assessment of patients with bipolar disorder should include investigation of exposure to childhood trauma in order to determine appropriate therapeutic strategies.

**Disclosure of Interest:**

None Declared

